# Dynamic ultrasound enables quantitative assessment of medial knee instability: A scoping review

**DOI:** 10.1002/jeo2.70606

**Published:** 2026-01-08

**Authors:** Paulo Roberto de Queiroz Szeles, Leonardo Addêo Ramos, André Fukunishi Yamada, Moisés Cohen, Mark Sayers, Benno Ejnisman

**Affiliations:** ^1^ Department of Orthopedics and Traumatology, Escola Paulista de Medicina Federal University of São Paulo (UNIFESP) São Paulo São Paulo Brazil; ^2^ School of Health, Sports and Exercise Science University of the Sunshine Coast (USC) Sunshine Coast Queensland Australia

**Keywords:** diagnostic reliability, medial knee instability, musculoskeletal ultrasound, valgus stress test

## Abstract

**Purpose:**

To map the scientific literature on the use of dynamic ultrasound in the assessment of medial knee instability.

**Methods:**

Primary (clinical or experimental) and secondary studies that used dynamic ultrasound to assess medial knee opening were included. There were no restrictions on language or date. Sources of information: The searches were conducted in the PubMed/MEDLINE, Scopus, Web of Science, Embase and CINAHL databases.

**Data selection and extraction:**

The process of data selection and extraction was conducted by two independent reviewers. The information was organised into thematic tables and a conceptual matrix was developed based on the components of population, concept, and context.

**Results:**

Ten studies were included: two biomechanical, six clinical, and two reviews. Ultrasound demonstrated good reliability in measuring medial opening and distinguishing between injured and normal knees. Heterogeneity was observed in the stress protocols, evaluation angles, units of measurement, and anatomical points.

**Conclusions:**

Dynamic ultrasound presents consolidated clinical potential in the assessment of medial knee instability. Standardisation of methods and additional clinical validation are necessary.

**Level of Evidence:**

Not applicable.

**Abbreviations**
ACL: anterior cruciate ligament
DUS: dynamic ultrasound
MCL: medial collateral ligament
MRI: magnetic resonance imaging
PCC: population, concept, and context

## INTRODUCTION

Medial knee instability, often resulting from medial collateral ligament (MCL) injuries, represents a significant diagnostic challenge in clinical and surgical practice, especially in acute cases, combined injuries, and the postoperative context. Traditionally, the assessment of ligament stability is performed through manual clinical tests, supplemented, when necessary, by imaging methods such as magnetic resonance imaging (MRI) or stress radiography [8, 9]. However, these approaches present limitations. MRI does not always reflect ligament functionality under load, while X‐ray depends on specific equipment and the application of standardised mechanical force.

Dynamic ultrasound (DUS) performed under controlled valgus stress allows direct observation of the MCL response to load, providing objective data on medial joint opening, ligament deformation and biomechanical behaviour [2, 15]. It should be noted that valgus stress primarily evaluates the superficial medial collateral ligament, although the deep MCL and posteromedial corner structures may also contribute to medial knee stability. Despite its potential, DUS use still lacks standardisation and formal recognition in diagnostic algorithms for medial instability [16]. The existing literature is fragmented, consisting of studies with diverse designs, variable techniques, and inconsistent terminologies. In addition, although biomechanical investigations have been carried out with cadavers, clinical studies, and secondary reviews, there is no clear consolidation of the methods, parameters used, or clinical cutoff values [4, 5].

Given the complexity of using DUS as a diagnostic tool, a traditional systematic review focused exclusively on efficacy or accuracy would not be appropriate. The exploratory nature and wide dispersion of the evidence indicate the necessity for a structured mapping of existing approaches, which justifies the adoption of the scoping review [7, 17]. This approach allows for the identification of available study types, describes how DUS has been used, outlines the technical parameters and clinical contexts adopted, and highlights persisting knowledge gaps.

In order to support future standardisations and methodological advancements, the purpose of this scoping review was to arrange and elucidate the existing body of evidence regarding the use of DUS in evaluating medial knee instability. It was postulated that DUS is a valid and underutilized technique supported by clinical and biomechanical data but lacking formal integration into diagnostic pathways and protocol standardisation.

## METHODS

### Protocol and registration

This scoping review was developed based on the methodological guidelines of the Joanna Briggs Institute following the Preferred Reporting Items for Systematic Reviews and Meta‐Analyses extension for Scoping Reviews framework [17].

### Eligibility criteria

The population, concept and context (PCC) framework was applied to define the eligibility criteria for this review, as follows: Population (P): human individuals with medial knee instability, as well as healthy knees used as a control group in comparative studies. Cadaveric models and validated biomechanical simulations that reproduce the condition of medial ligament instability were also included; Concept (C): use of DUS as a diagnostic tool for medial knee instability, either in isolation or in comparison with other recognised methods, such as MRI (conventional or dynamic), stress radiography, standardised clinical examinations, or arthroscopy surgery. The focus was on primary ligament injuries, both acute and chronic, and Context (C): studies conducted in clinical, academic, or experimental settings, including hospitals, imaging centres and anatomical and biomechanical laboratories.

In this context, studies falling into at least one of the following methodological categories were included: (i) diagnostic accuracy studies comparing DUS with accepted reference methods; (ii) clinical observational studies (cross‐sectional, cohort, or case‐control) investigating the application or performance of DUS; (iii) experimental studies with cadaveric models or validated biomechanical simulations exploring the behaviour of medial ligaments during dynamic tests; and (iv) systematic reviews with a compatible scope. Case reports, technical comments, or other forms of non‐systematic literature were also considered if they provided relevant information for the practice of DUS in assessing medial knee instability and allowed for clear and systematic data extraction. The inclusion of these documents is in accordance with the exploratory nature of scoping reviews [7, 17]. Studies that exclusively used static ultrasound, investigations focused on non‐ligamentous structures (menisci, cartilage or bone) unless directly associated with medial instability, and documents that do not allow the extraction of relevant minimum data were excluded.

### Sources of information

A thorough search was conducted in PubMed/MEDLINE, Scopus, Web of Science, Embase, and CINAHL. These databases were selected for their complementary indexing, multidisciplinary scope and significance in evidence‐based health research, as endorsed by recent recommendations for improving search strategies in systematic reviews [7]. Google Scholar was used to identify grey literature materials, such as dissertations, theses, and academic reports. A manual search of the reference lists of the included studies was performed to identify additional works. No restrictions were applied regarding language, publication date or editorial status.

### Search strategy

The search strategy was developed using controlled descriptors and text words related to DUS, medial knee instability, and ligamentous structures, including “ultrasound” [Title/Abstract] OR “ultrasonography” [Title/Abstract] OR “sonography” [Title/Abstract] OR “diagnostic imaging” [MeSH Terms]) AND (“knee” [Title/Abstract] OR “knee joint” [Title/Abstract]) AND (“medial collateral ligament” [Title/Abstract] OR “MCL” [Title/Abstract] OR “medial ligament” [Title/Abstract] OR “medial knee instability” [Title/Abstract]) AND (“dynamic” [Title/Abstract] OR “stress” [Title/Abstract] OR “valgus” [Title/Abstract]. Search strategies were developed in collaboration with a research librarian [3]. Each search was adapted to the specific syntax and indexing system of each database. The final search was completed on 6 May  2025. Detailed, database‐specific search strategies are provided in Supporting Information: S1.

### Selection of evidence sources

The selection of studies was conducted in three consecutive stages using Rayyan QCRI software, an online platform specifically developed to support systematic and scoping reviews. This software allows for blind, collaborative, and structured screening [14]. Initially, all retrieved references were imported into the software for automatic duplicate removal. Next, titles and abstracts were screened in a blind and parallel manner by two independent reviewers. Studies considered potentially eligible underwent a third stage of full‐text review to confirm inclusion criteria. Discrepancies between reviewers were resolved by consensus.

### Data extraction and variables

Data extraction and charting were performed independently by two reviewers using a standardised form aligned with the review objectives and the PCC framework. Discrepancies were addressed through discussion and consensus. The data extracted encompassed the subsequent categories: (i) Identification of the study—author, year, country and type of publication; (ii) Methodological characteristics include study design (cross‐sectional, cohort and experimental), population type (humans, cadavers and simulations), sample size, and inclusion/exclusion criteria. (iii) Information related to DUS includes modality, equipment utilised, patient positioning, type of transducer, stress manoeuvre, scan plane, target joint, and imaging parameters (e.g., B‐mode and Doppler). (iv) Anatomical structures assessed include the MCL, posteromedial corner and related medial components. (v) Comparative methods include MRI, stress radiography, clinical examination and the presence of a control group. (vi) Outcomes include diagnostic accuracy (sensitivity, specificity and predictive value), reproducibility (intra/interobserver) and clinical impact.

## RESULTS

### Selection of evidence sources

**Figure 1:**
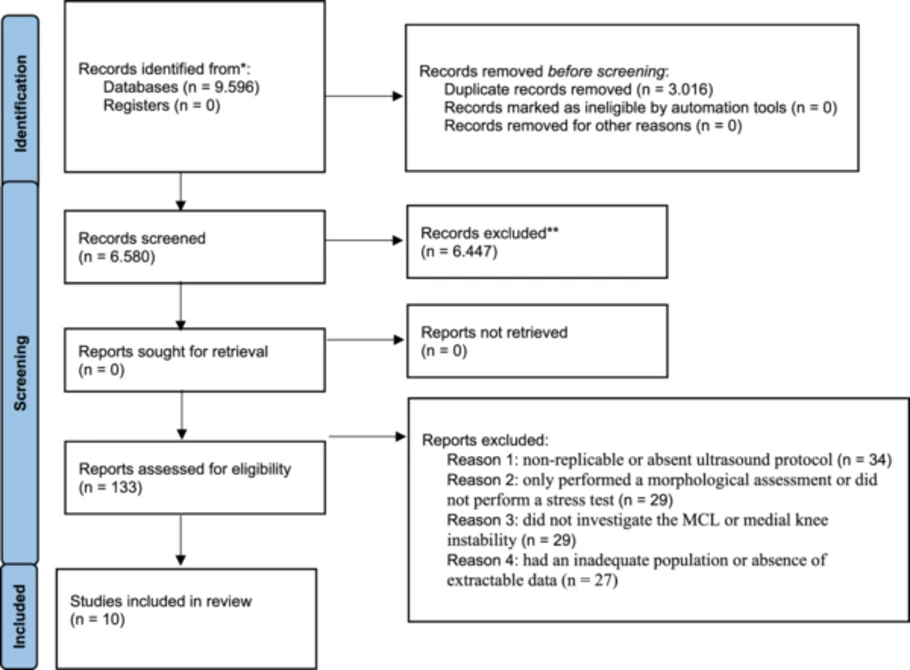
Flowchart of study selection according to PRISMA 2020 guidelines. The diagram illustrates the identification, screening, and inclusion process for studies assessing medial collateral ligament behaviour using dynamic ultrasound and other imaging modalities. DUS, dynamic ultrasound; MCL, medial collateral ligament.

After removal of duplicates, 6580 unique records were screened by title and abstract, originating from PubMed, Embase, Web of Science, CINAHL and Google Scholar, totalling 9596 initial entries. Duplicate removal was performed through automated processes on the management platform, eliminating 3016 entries. Following title and abstract screening, 6447 records were excluded for not meeting the PCC criteria. The main reasons for exclusion at this stage included use of static ultrasound only, evaluation of non‐ligamentous structures, a population without a diagnosis of medial instability or unsuitable publication type. Subsequently, 133 articles underwent full‐text evaluation; of these, 117 were excluded for reasons such as non‐replicable or absent DUS protocol, absence of stress testing, lack of investigation regarding the MCL or medial knee, or inadequate population/data. Ultimately, 10 studies met the eligibility criteria and were included in this review. The study selection process is summarised in Figure 1.

### Characteristics of the sources of evidence

The characteristics of each evidence source were extracted and organised according to study type, following predefined methodological criteria and the PCC strategy framework. The included studies were grouped into four primary categories: experimental biomechanical studies with cadavers (*n* = 2) [1, 16] clinical studies with diagnostic or functional assessment of the MCL (*n* = 6); [4, 5, 6, 8, 10, 11] and secondary technical‐clinical studies (systematic reviews and applied to practice; *n* = 2) [12, 13]. In total, 1465 persons and specimens were analysed, including 1452 clinical participants and 13 cadaveric knees; among comparative studies, 581 healthy controls and 871 patients with MCL injuries were included, with participant ages ranging from 13 to 101 years.

### Individual critical assessment of evidence sources

A critical evaluation of evidence sources was planned but not executed due to high methodological heterogeneity, which limited the applicability of standardised assessment tools. The analysed sample encompassed diverse designs, including cadaveric experimental studies, clinical observational studies, normative studies with healthy volunteers, systematic reviews and technical reports.

### Summary of results

The studies were classified into three major groups to facilitate comparative analysis and synthesis: (i) experimental biomechanical studies with cadaver specimens, (ii) clinical studies focused on medial joint opening or DUS accuracy and (iii) secondary and technical‐clinical studies, including systematic reviews and clinical guidance articles.

Cadaveric studies evaluated medial knee gap width using DUS under valgus stress. Bhimani et al. [1] examined eight cadaveric knees, applying 100 N of valgus force at 0° and 20° knee flexion, while Slane et al. [16] used 10 Nm of valgus load at approximately 20° knee flexion. Both studies demonstrated significant increases in medial gap width under stress. For example, Bhimani et al. [1] reported an increase from 6.6 ± 1.2 mm (unloaded) to 7.0 ± 1.1 mm (loaded) at 20° flexion, while Slane et al. [16] observed an increase from 8.7 ± 2.4 mm (unloaded) to 10.7 ± 2.2 mm under stress. The experimental characteristics of cadaveric and in situ studies investigating medial collateral ligament behaviour under valgus loading are summarised in Table 1.

**Table 1:** Summary of cadaveric and in situ experimental studies assessing medial collateral ligament behaviour using dynamic ultrasound.

Author (year)	Sample and assessesments	Medial opening	Findings
Bhimani et al. [1]	Cadaveric 8 knees (3 males, 5 females) evaluated the intact state and after selective transection of the sMCL and dMCLDUS (0 N and 100 N) at 0° and 20° knee flexion with a stress device	Unloaded 0°: Intact 6.5 (1.1) mm sMCL 7.3 (1.5) mm (Δ vs. Intact + 0.8 mm + 12.3%) dMCL 10.7 (2.2) mm (Δ vs. Intact + 4.2 mm + 64.6%) Loaded 0°: Intact 6.9 (1.1) mm sMCL 10.8 (2.4) mm (Δ vs. Intact + 3.9 mm + 56.5%) dMCL 14.8 (3.2) mm (Δ vs. Intact + 7.9 mm + 114.5%) Unloaded 20°: Intact 6.6 (1.2) mm sMCL 8.2 (2.0) mm (Δ vs. Intact + 1.6 mm + 24.2%) dMCL 11.3 (2.4) mm (Δ vs. Intact + 6.4 mm + 97%) Loaded 20°: Intact 7 (1.1) mm sMCL 10.9 (1.8) mm (Δ vs. Intact + 3.9 mm + 55.7%) dMCL 15.7 (2.4) mm (Δ vs. Intact + 8.7 mm + 124.3%)	At both 0° and 20° knee flexion, medial joint width increased with sequential MCL transection. At 20° with 100 N valgus stress, openings measured 7.0 ± 1.1 mm (intact), 10.9 ± 1.8 mm (sMCL), and 15.7 ± 2.4 mm (dMCL) (Δ = +8.7 mm; +124%, *p* < 0.001). A cutoff of 13.8 mm distinguished stable from unstable knees (AUC = 0.97; Se = 100%; Sp = 94.1%).
Slane et al. [16]	8 cadaveric (intact specimens) US data from the medial knee were collected and under knee valgus stress (10 Nm) in approximately 20°.	Medial gap width increased from 8.7 ± 2.4 mm (unloaded) to 10.7 ± 2.2 mm under 10 Nm valgus stress (Δ = 2.1 ± 0.7 mm; +24%)	Dynamic ultrasound demonstrated a consistent medial gap widening of approximately 2.1 mm ( + 24%) under valgus stress, accurately reflecting physiological joint laxity. Measurements showed excellent reliability and strong concordance with fluoroscopic assessment.

*Note*: This table presents experimental investigations that analysed MCL biomechanics using cadaveric or in‐situ knee models under different valgus loading conditions. The studies provide quantitative evidence of medial joint space changes under intact and sectioned ligament states, supporting the reliability of dynamic ultrasound for biomechanical assessment.

Abbreviations: DUS, dynamic ultrasound; MCL, medial collateral ligament.

Clinical studies included assessments in both healthy volunteers and patients with confirmed MCL injuries. DUS demonstrated high sensitivity and specificity for MCL rupture diagnosis (e.g., 87% sensitivity and 96% specificity) in the study of Friedl and Glaser [4]. Comparative studies with MRI yielded sensitivity and specificity values of 67% and 83%, respectively. Quantitative thresholds were established, with a sonographical elongation threshold of 5 mm differentiating MCL ruptures. Lutz et al. [10] provided more comprehensive normative data for healthy knees, demonstrating that medial joint width increased from 5.7 ± 1.2 mm unloaded to 7.4 ± 1.4 mm under a standardised 15 dekanewton load at 0° flexion and from 6.1 ± 1.1 mm to 7.8 ± 1.2 mm at 30° flexion. The study revealed significant gender differences, with female subjects showing lower medial joint width in both unloaded and loaded conditions. Kleinbaum and Blankstein [8] documented distinct structural alterations, noting that injured MCL exhibited increased thickness at the proximal attachment (6.4 mm compared to 4.3 mm in normal subjects) and distal attachment (4.4 mm versus 3.1 mm in controls). Post‐treatment assessment studies have shown the effectiveness of ultrasound in evaluating treatment outcomes. Lutz et al. [11] demonstrated that MCL repair and non‐operative treatment yielded similar radiological outcomes, with side‐to‐side differences of 0.4 ± 1.5 mm in the repair group and 0.9 ± 1.1 mm in the non‐operative group at 0° flexion, showing no statistically significant differences between the two approaches. Table 2 summarises clinical investigations evaluating the diagnostic performance and methodological approaches of DUS for medial collateral ligament assessment.

**Table 2:** Summary of studies evaluating medial collateral ligament assessment using dynamic ultrasound.

Author (year)	Sample and assessments	Medial opening	Diagnostic accuracy	Findings
Lutz et al. [11]	Group 1 (MCL repair, *n* = 20) Group 2 (no MCL repair, *n* = 20). Stress device (≈150 N)	Group 1 in 0°: Δ 2.3 0° mm vs. Group 2 in 0°: 2.1 (±0.7) mm. Group 1 in 30°: Δ 2.3 (±1.3) mm vs. Group 2 in 30°: 2.1 (±1.1) mm. Side‐to‐side difference in 0°: Group 1 = 0.4 mm (±1.5) vs. Group 2 = 0.9 mm (±1.1) Side‐to‐side difference in 30°: Group 1 = 0.4 mm (±1.5) vs. Group 2 = 0.5 mm (±1.4)	–	Both the MCL‐repair and non‐repair groups exhibited a comparable increase in medial joint space width under valgus load at 0° and 30° of knee flexion, approximately 2.1–2.3 mm. The side‐to‐side differences were minimal (< 1 mm) and did not reach statistical significance. The repair of the MCL did not have a significant impact on the dynamic behaviour of the medial joint when compared to conservative treatment.
Kleinbaum and Blankstein [8]	23 patients (18 men, 5 woman) with MCL injury and 18 sex‐ and age‐matched healthy controls. Manual stress.	Injured: 6.1 mm (range, 4–8.5 mm) to 10.5 mm (range, 7.6–14.0) (Δ = 4.4 mm/+72.1%) Normal: 6.7 (range, 5–7.7 mm) to 9.6 mm (range, 7.7–10.4 mm) (Δ = 2.9 mm/+43.3%)	–	The integration of sonographic findings in medial collateral ligament (MCL) injury with real‐time sonography during the valgus stress test can enhance clinical diagnosis and accurately identify the specific location of isolated MCL injuries.
Gruber et al. [6]	42 patients with MCL injury and 80 health controls. Manual and valgus stress (≈150 N) at 20° of flexion.	Injured: Manual: 4.4 mm (±1.1) vs. Device: 4.0 mm (±1.0 mm). (Δ = 0.4 mm/−9.1%) Controls: Manual: 2.2 mm (±0.5) vs. Device: 2.5 mm (±0.7).(Δ = 0.3 mm/+13.6%) (Δ = 4.4 mm/+72.1%)	–	In cases of injured knees, the Telos device resulted in a marginally reduced medial joint opening compared to manual valgus stress (–0.4 mm, –9%), whereas in healthy controls, it resulted in a slightly increased opening ( + 0.3 mm, +13%). The minimal differences observed suggest comparable performance and an absence of clinically significant variation between manual and device‐applied loading methods.
Ghosh et al. [5]	9 patients with MCL tears: Grade 1 (mild stretching), Grade 2 (partial fibre disruption), Grade 3 (complete discontinuity or retraction). POCUS with patients examined with knee flexed 20°–30° (manual)	–	Sensitivity 67%, specificity 83%, PPV 67%, NPV 83% vs. MRI. Two MCL tears were detected only by POCUS, while one chronic MCL tear was seen only on MRI.	POCUS (6–15 MHz, 20°–30° flexion) detected MCL tears with 67% sensitivity and 83% specificity vs MRI; suitable as a rapid bedside tool for medial collateral ligament assessment.
Friedl et al. [4]	84 patients with acute MCL injury, examined perioperatively by DUS, clinical evaluation, and confirmed by arthroscopy was performed under normal and valgus stress, with the knee positioned at 20° flexion	Intact ligaments: 2.9 (±1.4) mm of elongation Partial ruptures: 5.2 (±1.3) mm of elongation Complete ruptures: 6.6 (±1.6) mm of elongation	A threshold >5 mm identified rupture with 87% sensitivity, 96% specificity, and a 94% positive predictive value compared to arthroscopy; performance superior to clinical examination alone	A medial joint opening exceeding 5 mm under valgus stress suggests an MCL rupture, demonstrating 87% sensitivity, 96% specificity, and a 94% positive predictive value when compared to arthroscopic confirmation. Dynamic ultrasound exhibited diagnostic performance that surpassed clinical examination alone and was comparable to assessment under anaesthesia, thereby confirming its reliability in detecting and grading MCL injuries.
Lutz et al. [10]	65 healthy volunteers (79 knees). 32 females and 33 males without prior knee injury. Both knees were examined in 14 participants, resulting in a total of 79 healthy joints evaluated. 0° and 30° knee flexion, under unloaded and loaded (≈150 N) conditions, applied via device stress.	Knee at 0° of flexion: increased from 5.7 (±1.2) mm (unloaded) to 7.4 (±1.4) mm (loaded) Δ = 1.7 (±1.0) mm, +29.8% Knee at 30° of flexion: increased from 6.1 (±1.1) mm (unloaded) to 7.8 (±1.2) mm (loaded) Δ = 1.7 ± (±0.9) mm, +29.8%	–	Under standardised valgus loading of 15 daN applied by a device, the medial joint width increased by approximately 1.7 mm (≈+28–30%) at both 0° and 30° of knee flexion. The findings from 79 healthy knees assessed via dynamic ultrasound indicate a consistent physiological medial joint opening and establish normative reference values for evaluating medial ligament laxity.

*Note*: This table compiles clinical investigations assessing MCL integrity and diagnostic performance of dynamic ultrasound across different loading conditions and patient populations. The included studies compare manual and device‐applied valgus stress and benchmark ultrasound findings against arthroscopy or MRI for diagnostic accuracy.

Abbreviations: DUS, dynamic ultrasound; MCL, medial collateral ligament; MRI, magnetic resonance imaging.

Key findings from imaging literature indicate that MRI sensitivity varies widely, whereas ultrasonography and stress radiography present narrower and more predictable ranges. Notably, MRI underestimated surgical instability grades in approximately 21% of cases, highlighting the limitations of morphological evaluation alone. The lack of standardised classification systems and methodological discrepancies across studies was evident, limiting the comparability of outcomes. Multimodal assessment, integrating anatomical and functional evaluation, was found to be essential for accurate clinical decision‐making. Evidence from secondary sources, including one systematic and one narrative review on imaging modalities for MCL injuries, is presented in Table 3.

**Table 3:** Secondary evidence syntheses reporting imaging findings and diagnostic accuracy for medial collateral ligament injuries.

Author (year)	Design and scope	Key evidence and diagnostic performance	Clinical implications and applications	Study limitations and quality assessment
Manske et al. [12]	Narrative review examining DUS as a diagnostic tool for MCL injuries with specific focus on rehabilitation applications. Reviews technical aspects of ultrasound imaging, sonographic appearance across injury grades, and comparison with other imaging modalities like MR	MSK‐US demonstrates comparable diagnostic accuracy to MRI for assessing medial knee injuries. Offers dynamic, real‐time imaging capabilities with high‐frequency linear transducers producing detailed soft‐tissue images. Diagnostic criteria based on ligament thickness, loss of normal fibrillar pattern, and presence of preligamentous fluid. Enhanced by ability to perform comparative assessments of affected and contralateral sides	Provides immediate feedback for treatment decisions and allows rehabilitation providers to tailor exercise programs, guide manual therapy techniques, and monitor ligament healing progression. Enables ultrasound‐guided interventions such as injections with greater accuracy and safety. Particularly valuable due to portability, cost‐effectiveness, and ability to visualise structures under applied stress	Notable challenges include operator dependency, steep learning curve, and variability in image interpretation. Comprehensive training and experience essential for maximising diagnostic utility. Despite advantages, MSK‐US not yet standard modality for MCL assessment, with stress radiography and MRI more commonly used
Meyer et al. [13]	Systematic literature review of 23 imaging studies examining MRI, ultrasonography, and stress radiography for MCL injury assessment. Analysed 808 injured and 294 control knees across multiple imaging modalities. Methodological quality assessed using QUADAS‐2 tool	MRI showed moderate to strong correlation with clinical findings (65%–92%) but underestimated instability grade in up to 21% of cases compared to surgical findings. Interobserver reliability ranged from substantial to almost perfect agreement (0.76–0.93) for MRI. Ultrasonography demonstrated high interobserver reliability with almost perfect agreement. Stress radiography showed almost perfect intraobserver reliability (0.96–0.99).	MRI highly sensitive in detecting MCL lesions compared to clinical examination but demonstrated inferior performance in identifying exact lesion location compared to surgical findings. Ultrasonography represents cost‐efficient, radiation‐free dynamic imaging method but rarely used in clinical practice. Stress radiography findings correlate with surgical findings but clinical correlations missing in literature.	QUADAS‐2 assessment showed low risk of bias for patient selection and index testing but increased risk for reference standard and flow/timing. Large heterogeneity of findings made studies difficult to compare and unfeasible for meta‐analysis. Paucity of high‐quality literature reliably comparing different imaging modalities based on validated gradings. Study quality limited by source literature quality.

*Note*: This table summarises secondary studies, including one systematic and one narrative review, that synthesised evidence on imaging modalities for MCL injury diagnosis. It highlights the diagnostic performance of dynamic ultrasound compared with MRI and stress radiography, discussing methodological limitations and clinical implications.

Abbreviations: DUS, dynamic ultrasound; MCL, medial collateral ligament; MRI, magnetic resonance imaging; QUADAS‐2, Quality Assessment of Diagnostic Accuracy Studies – version 2.

Table 4 presents a conceptual matrix with the aim of mapping the correspondence of each concept with the central components of the review question and with the 10 included studies. The analysis of the studies included in the conceptual matrix reveals that all of them align fully with the core components of the review question.

**Table 4:** Conceptual matrix mapping the alignment of core review components with included studies.

Author (year)	Population	Concept	Context	Direct contribution to the objectives of the review
Bhimani et al. [1]	Yes. Cadaveric with simulation of MCL injury	Yes. Assessment of medial opening with and without load	Yes. Experimental biomechanical study	Contributes with differential values in isolated ligaments
Slane et al. [16]	Yes. Cadaveric with simulated ligament tension	Yes. Assessment of ligament strain by ultrasound	Yes. Experimental biomechanical study	Shows structural behaviour of the MCL under stress
Lutz et al. [11]	Yes. Individuals without ligament injury	Yes. Variation in medial opening with Telos	Yes. Normative cross‐sectional clinical study	Establishes reference values for medial opening
Kleinbaum et al. [8]	Yes. Patients with and without MCL injury	Yes. Medial gap compared with manual loading	Yes. Comparative clinical study	Demonstrates difference between injured and normal knees
Gruber et al. [6]	Yes. Patients with unilateral MCL rupture	Yes. Side‐by‐side assessment by ultrasound	Yes. Observational clinical study	Establishes direct comparison between injured and contralateral knees
Ghosh et al. [5]	Yes. Patients with clinical suspicion of injury	Yes. Point‐of‐care US (POCUS) diagnosis	Yes. Diagnostic clinical study	Analyses diagnostic accuracy of US compared to MRI
Friedl and Glaser [4]	Yes. Patients with ligament injuries	Yes. Medial gap measurement under ultrasound stress	Yes. Diagnostic clinical study	Defines clinical cut‐off point and compares with arthroscopy
Lutz et al. [11]	Yes. Post‐operative ACL + MCL patients	Yes. Assessment of stability with Telos and US	Yes. Postoperative clinical study	Assesses stability after surgical intervention
Manske et al. [13]	Yes. Clinical and rehabilitation discussion	Yes. Narrative review of dynamic US	Yes. Clinical secondary review	Integrates clinical use and recommendations in rehabilitation
Meyer et al. [13]	Yes. Various clinical populations	Yes. Systematic assessment of diagnostic accuracy of US	Yes. Systematic secondary review	Assesses reliability and methodological limitations

*Note*: This table maps the alignment between each included study and the key conceptual domains of the review, including population, concept, and context. The matrix demonstrates that all studies explicitly contribute to the core objectives of the systematic review, supporting its conceptual comprehensiveness.

A chronological analysis of the included studies demonstrates a clear methodological evolution in the use of DUS for assessing medial knee instability. Earlier studies applied DUS in an exploratory manner within diagnostic clinical contexts, often utilising poorly standardised protocols and relying primarily on manual stress application [4, 6]. Despite these limitations, these pioneering works introduced objective measures, such as clinical cutoff points based on medial joint opening measured in millimetres. From 2007 onward, study designs began to incorporate structured comparisons between injured and uninjured knees, resulting in improved methodological rigour and greater control over confounding variables [8]. A significant qualitative advance was observed from 2017, marked by the introduction of controlled cadaveric biomechanical models and the adoption of advanced imaging technologies, including speckle tracking and ligament strain analysis [1, 5, 16]. In the past decade, DUS has been increasingly utilised in specific clinical contexts such as postoperative evaluation of ligament and joint stability [11], as well as in rehabilitation settings [12]. The technique has also become the subject of systematic reviews [13], reflecting its growing clinical and research significance. This methodological progression underscores the transformation of DUS from an exploratory diagnostic tool to a more structured and potentially standardised method, with the capacity to consolidate its role in the routine assessment of medial knee instability.

## DISCUSSION

This scoping review systematically mapped and synthesised the existing evidence on the use of DUS for assessing medial knee instability. The studies were categorised as cadaveric, clinical, and secondary sources, reflecting a growing methodological and practical interest in this imaging modality. This rationale aligns with the evaluation of the anterior cruciate ligament, where subjective assessments like the Lachman and pivot shift tests are complemented by quantitative measures of translation and acceleration. DUS facilitates the substitution of subjective valgus stress testing with an objective and quantitative assessment of medial instability. DUS offers specific advantages over conventional imaging methods. Unlike MRI, which provides static anatomical data, ultrasound enables real‐time dynamic evaluation under stress at the point of care. Ghosh et al. [5] highlighted that ultrasound is a non‐invasive and cost‐effective bedside procedure that facilitates real‐time diagnosis and early management of injuries, offering significant time advantages for both patients and physicians. Stress radiography measures medial gapping; however, it requires calibrated instruments, exposes patients to radiation, and may demonstrate inconsistencies in implementation. In contrast, ultrasound is non‐radiative, portable, and cost‐efficient.

The evidence indicates that DUS assessment of MCL injuries has transitioned from an experimental diagnostic tool to a clinically validated method, complete with established normative values and diagnostic thresholds. Friedl and Glaser [4] reported that DUS exhibited 87% sensitivity and 96% specificity in the diagnosis of medial collateral ligament ruptures, outperforming clinical examination alone significantly. Ghosh et al. [5] found that ultrasound demonstrated 67% sensitivity and 83% specificity in detecting MCL tears when compared to MRI.

Friedl and Glaser [4] determined that a sonographical elongation threshold of 5 mm effectively distinguishes MCL ruptures. Intact ligaments exhibit an elongation of 2.9 ± 1.4 mm, partial ruptures show 5.2 ± 1.3 mm, and complete ruptures demonstrate 6.6 ± 1.6 mm. This threshold demonstrated a positive predictive value of 94% for identifying ruptures when compared to arthroscopy.

Lutz et al. [10] presented normative data for healthy knees, indicating that medial joint width increased from 5.7 ± 1.2 mm in an unloaded state to 7.4 ± 1.4 mm under a standardised 15 dekanewton load at 0° flexion, and from 6.1 ± 1.1 mm to 7.8 ± 1.2 mm at 30° flexion. The research revealed notable gender disparities, as female participants exhibited reduced medial joint width under both unloaded and loaded conditions. Kleinbaum and Blankstein [8] demonstrated that ultrasound is capable of detecting structural changes, revealing that injured MCLs exhibit greater thickness compared to normal ligaments. The proximal medial collateral ligament (MCL) measured 6.4 mm, in contrast to 4.3 mm in normal subjects, while the distal MCL averaged 4.4 mm compared to 3.1 mm in controls. The integration of sonographic findings with real‐time valgus stress testing enhances clinical diagnosis and accurately identifies specific injury sites.

Lutz et al. [11] validated the utility of ultrasound in post‐treatment assessment, showing that MCL repair and non‐operative treatment yielded similar radiological outcomes. Side‐to‐side differences were minimal, with the repair group exhibiting 0.4 ± 1.5 mm differences at both 0° and 30° flexion. In contrast, the non‐operative group demonstrated 0.9 ± 1.1 mm at 0° and 0.5 ± 1.4 mm at 30°, with no statistically significant differences observed between the groups. This evidence supports the use of ultrasound in monitoring treatment outcomes and confirming the restoration of medial knee stability.

The variability in flexion angles, stress application techniques, and loading protocols significantly restricts comparability and obstructs the establishment of universal thresholds, thereby impeding the development of standardised clinical guidelines. Friedl and Glaser [4] highlighted the operator dependency of ultrasound, underscoring the significant learning curve and reliance on the training, skill, and experience of the operator. The absence of standardised protocols can lead to inconsistent outcomes in the evaluation of knee pathology.

Friedl and Glaser [4] concluded that sonography should be routinely employed as a primary diagnostic tool following clinical examination due to its cost‐effectiveness, absence of side effects, lack of anaesthesia requirement, ability for repeated use and high sensitivity and specificity.

The rehabilitation‐focused perspective highlights practical implementation barriers, notably operator dependency and a steep learning curve, which restrict the adoption of musculoskeletal ultrasound despite its theoretical benefits. The systematic analysis reveals methodological concerns, such as variability in study designs and quality deficiencies in the source literature. The findings indicate that the field necessitates not only technological progress but also essential standardisation of protocols, training programs, and classification systems to fully leverage the potential of existing imaging modalities in clinical practice.

Future research must focus on the standardisation of stress protocols, the establishment of comprehensive normative data across various populations, and the validation of DUS against gold‐standard diagnostics through prospective, comparative studies. The development of clinical guidelines and structured training programs is crucial for promoting broader implementation and ensuring reliability. Ghosh et al. [5] proposed that increasing sample sizes and recruiting participants from diverse settings, including emergency departments, may enhance the robustness of future observational studies.

## CONCLUSIONS

DUS has been shown to be a promising and accessible tool for the assessment of medial knee instability. Its application in biomechanical, clinical, and surgical settings demonstrates its versatility, while the findings of studies suggest good accuracy and reliability in different contexts. However, the lack of standardisation in evaluation protocols limits its evolution into a standard reference method.

Future recommendations include employing standardised designs and methods, in which it is possible to systematise the evaluation angles, the applied loads, and the presentation of the units of measurement among the studies. The validation of the instrument in diagnostic accuracy studies in the context of medial knee instability should also be a priority.

The current mapping provides a conceptual basis for the construction of clinical guidelines and standardised usage protocols of DUS in the diagnosis and monitoring of medial knee instability.

## AUTHOR CONTRIBUTIONS

Conceptualisation: Paulo Roberto de Queiroz Szeles. Methodology and Search Strategy: Paulo Roberto de Queiroz Szeles, Leonardo A. Ramos. Screening and Data Extraction: André F. Yamada, Leonardo A. Ramos. Data Analysis and Synthesis: Paulo Roberto de Queiroz Szeles, André F. Yamada. Manuscript writing/Original Draft: Paulo Roberto de Queiroz Szeles. Manuscript Review and Editing: All authors read and approved the final version of the manuscript.

## CONFLICT OF INTEREST STATEMENT

The authors declare no conflicts of interest.

## ETHICS STATEMENT

This article is a scoping review and did not involve human participants or animal subjects; therefore, ethical approval was not required. The protocol was registered on the Open Science Framework (OSF) platform before the start of the data collection and analysis stage (Identifier: DOI 10.17605/OSF.IO/FR7G2).

## Supporting information

Questionnaire_for_dermatologists

## Data Availability

All data extracted and synthesised during this study are available from the corresponding author upon reasonable request.
