# Supplementary material for: Dynamic ultrasound enables quantitative assessment of medial knee instability: A scoping review
Source: J Exp Orthop. 2026 Jan 8;13(1):e70606. doi: 10.1002/jeo2.70606 (PMC12780856; doi:10.1002/jeo2.70606)
Supplement: Supplementary file 1 — Questionnaire_for_dermatologists [file JEO2-13-e70606-s001.pdf]

Appendix A: Comprehensive Search Strategies for Each Database

In PubMed, the following search strategy was employed: ("Knee Joint"[Mesh] OR "knee instability"[tiab] OR "medial knee instability"[tiab]) AND ("Ultrasonography"[Mesh] OR "ultrasound imaging"[tiab] OR "musculoskeletal ultrasound"[tiab]) AND ("Medial Collateral Ligament"[Mesh] OR "medial collateral ligament"[tiab] OR "MCL"[tiab]).

In Embase, the modified query was: ('knee instability'/exp OR 'medial knee instability':ti,ab) AND ('ultrasonography'/exp OR 'ultrasound imaging':ti,ab OR 'musculoskeletal ultrasound':ti,ab) AND ('medial collateral ligament'/exp OR 'medial collateral ligament':ti,ab OR 'MCL':ti,ab).

In Web of Science, the following search phrases were employed: TS=("knee instability" OR "medial knee instability") AND TS=("ultrasound imaging" OR "musculoskeletal ultrasound") AND TS=("medial collateral ligament" OR "MCL").

The CINAHL search comprised: ("knee instability" OR "medial knee instability") AND ("ultrasound" OR "ultrasonography" OR "musculoskeletal ultrasound") AND ("medial collateral ligament" OR "MCL").

The streamlined query for Google Scholar was allintitle: "knee instability" "ultrasound" "medial collateral ligament".
